# VALIDATION OF A BRIEF SCREEN TO IDENTIFY PERSONS WITH DEMENTIA AT RISK FOR BEHAVIORAL DISTURBANCE

**DOI:** 10.1093/geroni/igz038.3366

**Published:** 2019-11-08

**Authors:** Tracy L Evans, Amber Amspoker, Mark E Kunik, Srijana Shrestha

**Affiliations:** 1 Baylor College of Medicine, Houston, Texas, United States; 2 Wheaton College, MA, Norton, United States

## Abstract

Per current guidelines, clinical assessment of persons with dementia (PWD) should include potential causes of behavioral and psychiatric problems including pain, depression, and caregiver-patient relationship quality. Many validated assessment tools are available; however, administering a battery of instruments is not practical in most clinical settings. Objectives of this secondary analysis are to 1) evaluate the construct validity of brief screens (1-3 questions each) for pain, depression, and relationship strain by examining their associations with validated measures (Geriatric Depression Scale, Modified Philadelphia Pain Scale, Zarit Burden Interview, Mutuality Scale) and medication use and 2) evaluate the predictive validity of each individual screen and the screens as a set (number positive) by examining their associations with frequency of disruptive behaviors on the Revised Memory and Behavior Problem Checklist. PWDs (n=228) were included in the original trial if the PWD or the caregiver endorsed one or more of the three screens. There was evidence of good convergent and discriminate validity for each individual screen (p’s < 0.01). Although only the relationship screen was individually associated with frequency of disruptive behaviors (p < 0.00010), the total number of screens endorsed was positively associated with this frequency (F (2,225) = 5.50, p = 0.005). In this sample, the brief screening questions showed good construct and predictive validity. Further studies are needed to determine if they can be used to identify patients with depression, pain, and/or caregiver-patient relationship problems in the clinical setting.

